# Histopathological response to chemotherapy and survival of mucinous type gastric cancer

**DOI:** 10.1093/jnci/djae227

**Published:** 2024-09-14

**Authors:** Irene A Caspers, Astrid E Slagter, Pauline A J Vissers, Martha Lopez-Yurda, Laurens V Beerepoot, Jelle P Ruurda, Grard A P Nieuwenhuijzen, Suzanne S Gisbertz, Mark I van Berge Henegouwen, Henk H Hartgrink, Danny Goudkade, Liudmila L Kodach, Johanna W van Sandick, Marcel Verheij, Rob H A Verhoeven, Annemieke Cats, Nicole C T van Grieken

**Affiliations:** Department of Gastrointestinal Oncology, Netherlands Cancer Institute, Antoni van Leeuwenhoek, Amsterdam, the Netherlands; Department of Pathology, Amsterdam University Medical Center, Cancer Center Amsterdam, Amsterdam, the Netherlands; Department of Radiation Oncology, Netherlands Cancer Institute, Antoni van Leeuwenhoek, Amsterdam, the Netherlands; Department of Research and Development, Netherlands Comprehensive Cancer Organization (IKNL), Utrecht, the Netherlands; Department of Surgery, Radboud University Medical Center, Nijmegen, the Netherlands; Department of Biometrics, Netherlands Cancer Institute, Antoni van Leeuwenhoek, Amsterdam, the Netherlands; Department of Oncology, Elisabeth Two Cities Hospital, Tilburg, the Netherlands; Department of Surgical Oncology, University Medical Center Utrecht, Utrecht, the Netherlands; Department of Surgery, Catharina Hospital, Eindhoven, the Netherlands; Department of Surgery, Amsterdam University Medical Center, University of Amsterdam, Amsterdam, the Netherlands; Cancer Treatment and Quality of Life, Cancer Center Amsterdam, Amsterdam, the Netherlands; Department of Surgery, Amsterdam University Medical Center, University of Amsterdam, Amsterdam, the Netherlands; Cancer Treatment and Quality of Life, Cancer Center Amsterdam, Amsterdam, the Netherlands; Department of Surgical Oncology, Leiden University Medical Center, Leiden, the Netherlands; Department of Pathology, Zuyderland Medical Center, Sittard-Geleen, the Netherlands; Department of Pathology, Netherlands Cancer Institute, Antoni van Leeuwenhoek, Amsterdam, the Netherlands; Department of Surgical Oncology, Netherlands Cancer Institute, Antoni van Leeuwenhoek, Amsterdam, the Netherlands; Department of Radiation Oncology, Netherlands Cancer Institute, Antoni van Leeuwenhoek, Amsterdam, the Netherlands; Department of Radiation Oncology, Radboud University Medical Center, Nijmegen, the Netherlands; Department of Research and Development, Netherlands Comprehensive Cancer Organization (IKNL), Utrecht, the Netherlands; Cancer Treatment and Quality of Life, Cancer Center Amsterdam, Amsterdam, the Netherlands; Department of Medical Oncology, Amsterdam University Medical Centers, Amsterdam, the Netherlands; Department of Gastrointestinal Oncology, Netherlands Cancer Institute, Antoni van Leeuwenhoek, Amsterdam, the Netherlands; Department of Pathology, Amsterdam University Medical Center, Cancer Center Amsterdam, Amsterdam, the Netherlands

## Abstract

**Background:**

Data on the clinicopathological characteristics of mucinous gastric cancer (muc-GC) are limited. This study compares the clinical outcome and response to chemotherapy between patients with resectable muc-GC, intestinal (int-GC), and diffuse (dif-GC) gastric cancer.

**Methods:**

Patients from the D1/D2 study or the CRITICS trial were included in exploratory surgery-alone (SA_test_) or chemotherapy test (CT_test_) cohorts. Real-world data from the Netherlands Cancer Registry on patients treated between with surgery alone (SA_validation_) and receiving preoperative chemotherapy with or without postoperative treatment (CT_validation_) were used for validation. Histopathological subtypes were extracted from pathology reports filed in the Dutch Pathology Registry and correlated with tumor regression grade (TRG) and relative survival (RS).

**Results:**

In the SA_test_ (n = 549) and SA_validation_ (n = 8062) cohorts, muc-GC patients had a 5-year RS of 39% and 31%, similar to or slightly better than dif-GC (43% and 29%, *P* = .52 and *P* = .011), but worse than int-GC (55% and 42%, *P* = .11 and *P* < .001). In the CT_test_ (n = 651) and CT_validation_ (n = 2889) cohorts, muc-GC showed favorable TRG (38% and 44% (near-) complete response) compared with int-GC (26% and 35%) and dif-GC (10% and 28%, *P* < .001 and *P* = .005). The 5-year RS in the CT_test_ and CT_validation_ cohorts for muc-GC (53% and 48%) and int-GC (58% and 59%) was significantly better compared with dif-GC (35% and 38%, *P* = .004 and *P* < .001).

**Conclusion:**

Recognizing and incorporating muc-GC into treatment decision-making of resectable GC can lead to more personalized and effective approaches, given its favorable response to preoperative chemotherapy in relation to int-GC and dif-GC and its favorable prognostic outcomes in relation to dif-GC.

Gastric cancer (GC) is most commonly classified according to its histopathological appearance using the Lauren classification (1). This classification system distinguishes 2 primary subtypes of GC that differ both morphologically and clinically: the intestinal type and diffuse type. Patients with intestinal type GC (int-GC) have a more favorable prognosis and a better response to chemotherapeutic treatment compared with those with diffuse type GC (dif-GC) (2-6). However, there is a subset of GCs that does not fit into the Lauren classification. This issue was recently addressed by Smyth et al. showing the overlap of the different GC classification systems (7). The group of “other” subtypes that do not fit in Lauren’s classification includes, among others, the mucinous type GC (muc-GC).

Muc-GC is characterized by the presence of larger than 50% extracellular mucus. It was first described in the World Health Organization (WHO) classification in the year 1990 (8) and accounts for approximately 3% to 7% of all gastric adenocarcinomas (9-12). Several retrospective studies have demonstrated that muc-GC is associated with more advanced tumor stages and, consequently, inferior survival when compared with non-mucinous subtypes (13-17).

Data on the response to preoperative chemotherapy in muc-GC are lacking. In other gastrointestinal cancers, this has been more extensively studied (18-20). Patients with mucinous adenocarcinomas of the colorectum, for example, have an inferior response to preoperative chemoradiotherapy than patients with other subtypes (19, 20). In esophageal adenocarcinomas, on the other hand, patients with mucinous type tumors showed improved survival when treated with preoperative chemotherapy and surgical resection than those treated with surgery alone (18). Whether or not the prognosis of patients with muc-GC will improve after preoperative chemotherapy, similar to esophageal adenocarcinomas, or if it will continue to be inferior to non-mucinous phenotypes, similar to the prognosis of mucinous colorectal cancer, remains unclear. The aim of this study was, therefore, to investigate the histopathological response to chemotherapy and survival of muc-GC compared with int-GC and dif-GC.

## Methods

### Patients

**Figure 1.**
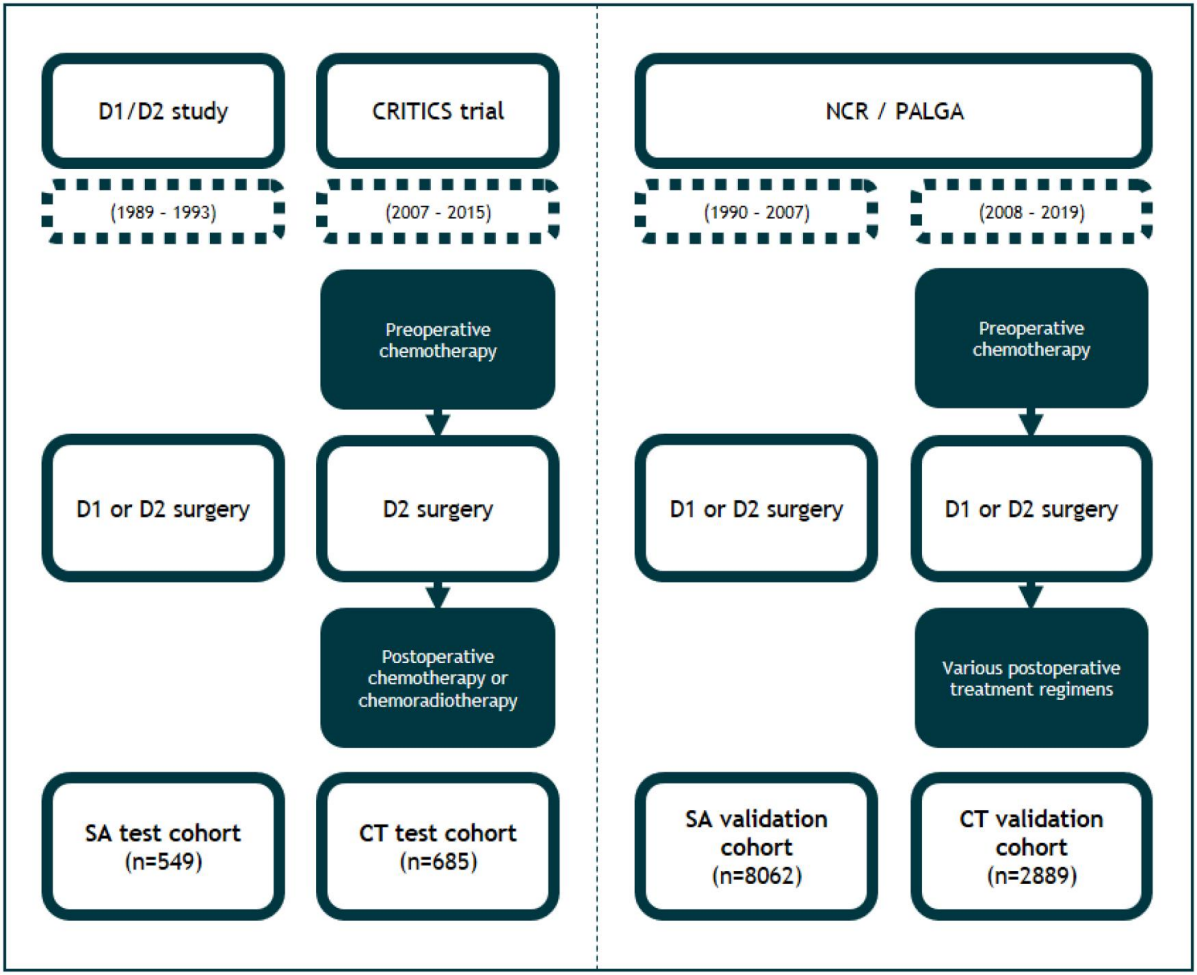
Overview of patient cohorts. NCR = Netherlands Cancer Registry; PALGA = Dutch Pathology Registry; SA = surgery alone; CT = chemotherapy.

Data of patients with resectable GC were assembled from 2 clinical trials for exploratory analyses: (a) patients included in the Dutch D1/D2 study as the surgery alone test cohort (SA_test_) and (b) patients included in the international CRITICS trial as the chemotherapy test cohort (CT_test_) (Figure 1). In the randomized controlled D1/D2 study, 711 patients were treated with (sub)total gastrectomy and either a D1 or D2 lymphadenectomy between 1989 and 1993 (21). No chemotherapy was administered in the D1/D2 study. In the CRITICS trial, 788 patients were randomized between 2007 and 2015 to receive preoperative chemotherapy followed by (sub)total gastrectomy and either postoperative chemotherapy or chemoradiotherapy (22). Histopathological subtypes of GC were centrally reviewed on resection specimens by an expert gastrointestinal (GI) pathologist. Histopathological subtypes were classified as intestinal, diffuse, mixed, mucinous and other type GC, or unknown.

For validation, a nationwide cohort (c) was assembled using clinical data of the Netherlands Cancer Registry (NCR) between 1990 and 2019 (Figure 1). The NCR is a nationwide registry including all newly diagnosed cancer patients in the Netherlands in which clinicopathological characteristics are obtained from medical records on a regular basis by data managers. Variables extracted from the NCR for the present study included age at diagnosis, sex, tumor localization, stage, treatment specifications, and survival status. Because collection of histopathological subtypes is not part of the regular data collection of the NCR, patient data were matched to the patients’ corresponding pathology reports filed by the Dutch Pathology Registry (PALGA). PALGA is a nationwide registry enrolling all histopathological diagnoses in the Netherlands (23).

All GC patients registered between January 1, 1990 and December 31, 2019 who underwent a (sub)total gastrectomy were included in this analysis. Patients were categorized into 2 time periods: the surgery alone validation cohort (SA_validation_), including all patients treated with surgery alone before the introduction of perioperative chemotherapy as standard of care treatment (1990-2007); and the chemotherapy validation cohort (CT_validation_), including patients after the introduction of perioperative chemotherapy (2008-2019). Patients who did not receive any preoperative chemotherapy in the chemotherapy period were excluded. Patients who died perioperatively or were lost to follow-up the day after surgery were excluded from the survival analyses. Furthermore, tumors of mixed type, other type, or unknown histology were outside the scope of this study and therefore excluded from further analyses.

### Histopathology of validation cohorts

In the validation cohorts, the pathology reports of resection specimens or—in the case of a complete response to chemotherapy—pretreatment biopsies were used to specify a tumor’s histopathological subtype. Determination of the histopathological subtype was assessed using an adapted version of the method described previously by van der Kaaij et al. (6). In brief, a syntax was developed to classify gastric tumors as either intestinal type, diffuse type, mixed type, mucinous type, or unknown using keywords specific for each histopathological subtype (Supplementary Table 1, available online). To validate the syntax, 2 researchers (IAC and AES) manually determined the histopathological subtype on a subset of pathology reports. To detect a 95% overall agreement in histopathological subtype determined by the manual method and by the syntax with 90% power, an evaluation of 328 pathology reports was needed (55% intestinal, 36% diffuse and 9% mucinous), including 8 expected unevaluable. This resulted in a 96% overall proportion of agreement in int-GC, dif-GC, and muc-GC between the manual method and the syntax. All tumors that were manually classified as muc-GC were likewise identified by the syntax (sensitivity 1.0, specificity 0.97). For the CT_validation_ cohort, tumor regression grade according to Mandard (TRG) was extracted from the pathology files using a similar syntax (Supplementary Table 2, available online) (24). Patients with a pT0 tumor stage were categorized as TRG1 regardless of whether TRG was described within the pathology report, resulting in an overrepresentation of TRG1 across all histological subtypes in the CT_validation_ cohort.

### Outcomes

Clinicopathological characteristics and patients’ survival were analyzed for int-GC, dif-GC, and muc-GC separately in each cohort and time period and included age at diagnosis, sex, and tumor localization, type of surgery, administration of pre- or postoperative radiotherapy, administration and type of administered pre- or postoperative chemotherapy (available from 2015 for the CT_validation_ cohort), pathological tumor stage (pT), pathological lymph node stage (pN), and—for the chemotherapy cohorts—TRG. pT stages were updated to the 8th edition AJCC/UICC TNM classification (Supplementary Table 3, available online) (25). pT stages in the validation cohorts that could not be updated to the 8th edition TNM classification—for example, pT2 tumors scored before the year 2003—were reported as unknown. Relative survival (RS) according to the Pohar Perme method was calculated for all patients from time since surgery until death from any cause or last follow-up (26). The Pohar Perme is a method to estimate the cancer-specific survival, when the cause of death is unknown. It evaluates a patient’s impact on survival by considering the expected survival based on the mortality rate in a specific calendar year of the general Dutch population of the same sex and age. This method is widely used and can be regarded as a gold standard to estimate net survival (27).

### Statistical analysis

Differences in baseline and clinicopathological characteristics between muc-GC versus int-GC and dif-GC were pairwise tested using the Mann-Whitney *U* test for continuous variables and the χ^2^ test or Fisher exact test, when appropriate, for categorical data. Relative survival was estimated to adjust for sex and age differences. Muc-GC was used as a reference category. Multivariable relative excess risks of death (RER) were estimated with 95% confidence intervals (CI). Analyses were adjusted for sex, age at diagnosis, and year of diagnosis but not for clinical nor pathological tumor stage. Accurate staging can only be performed post-surgery on the resection specimen, but this can be influenced by potential downstaging resulting from a positive response to chemotherapy. In order to keep all study cohorts similar, correction for clinical and pathological factors was not performed on the surgery alone cohorts as well. Statistical significance was set at less than .05. All analyses were performed using SAS software version 9.4, R software version 4.2.1, and Stata software version 17.

## Results

###  

#### Surgery alone test cohort

**Figure 2.**
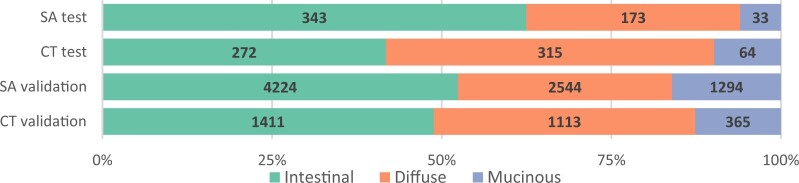
Distribution of histopathological subtypes per cohort. SA = surgery alone; CT = chemotherapy.

In total, 549 patients included in the D1/D2 study were included in the SA_test_ cohort. Among these were 343 (62%) intestinal, 173 (32%) diffuse, and 33 (6%) muc-GC patients (Figure 2). The median follow-up was 3.7 years (interquartile range [IQR] = 1.2–13.4). As shown in Table 1, patients with muc-GC had a significantly older age at diagnosis than patients with dif-GC, similar to int-GC. There were no significant differences in sexes. Information on tumor location was not available for the SA_test_ cohort. Gastrectomy with curative intent could be performed in 81% of muc-GC versus 93% of int-GC and 87% of dif-GC patients (*P* = .019 and *P* = .416). Advanced tumor stage (pT3-pT4) and lymph node metastases (pN) were more often diagnosed in muc-GC compared with int-GC (*P* = .007 and *P* = .01, respectively).

**Table 1.** Clinicopathological characteristics per cohort^a^

	Surgery alone test cohort					Chemotherapy test cohort					Surgery alone validation cohort					Chemotherapy validation cohort				
	Int-	Dif-	Muc-	*P*	*P*	Int-	Dif-	Muc-	*P*	*P*	Int-	Dif-	Muc-	*P*	*P*	Int-	Dif-	Muc-	*P*	*P*
	GC	GC	GC	Int vs muc	Dif vs muc	GC	GC	GC	Int vs muc	Dif vs muc	GC	GC	GC	Int vs muc	Dif vs muc	GC	GC	GC	Int vs muc	Dif vs muc
	(n = 343)	(n = 173)	(n = 33)			(n = 272)	(n = 315)	(n = 64)			(n = 4224)	(n = 2544)	(n = 1294)			(n = 1411)	(n = 1113)	(n = 365)		
**Median age**	67	60	66	*0.440*	*0.035*	65	59	63	*.046*	.010	71	67	70	*<0.001*	*<0.001*	67	63	66	*<0.001*	*0.029*
(IQR)	(60-74)	(52-69)	(59-72)			(58-71)	(50-66)	(56-69)			(64-78)	(57-74)	(61-77)			(59-72)	(54-70)	(60-70)		
**Sex**				*0.434*	*0.188*				*.054*	*.209*				*0.030*	*<0.001*				*<0.001*	*0.462*
Male	221 (64%)	78 (45%)	19 (58%)			210 (77%)	180 (57%)	42 (66%)			2882 (68%)	1366 (54%)	841 (65%)			1036 (73%)	606 (54%)	261 (72%)		
Female	122 (36%)	95 (55%)	14 (42%)			62 (23%)	135 (43%)	22 (34%)			1342 (32%)	1178 (46%)	453 (35%)			375 (27%)	507 (46%)	104 (28%)		
**Tumor location**																				
GEJ/Cardia	–	–	–			71 (26%)	24 (7%)	14 (22%)	*.099*	<.001	1001 (33%)	303 (19%)	306 (33%)	*0.160*	*<0.001*	448 (37%)	122 (15%)	124 (45%)	*<0.001*	*0.019*
Proximal	–	–	–			63 (23%)	59 (19%)	13 (20%)			98 (3%)	28 (2%)	29 (3%)			69 (5%)	28 (4%)	14 (5%)		
Middle	–	–	–			57 (21%)	119 (38%)	8 (12%)			427 (14%)	251 (16%)	105 (11%)			282 (23%)	229 (29%)	43 (15%)		
Distal	–	–	–			81 (29%)	113 (36%)	29 (45%)			1501 (50%)	1017 (63%)	485 (52%)			426 (35%)	404 (52%)	97 (35%)		
Unknown	–	–	–								1197	945	369			186	330	87		
**Curative surg.**				*0.019*	*0.416*				*.883*	*.460*										
Yes	318 (93%)	150 (87%)	26 (81%)			236 (88%)	259 (83%)	56 (89%)			–	–	–	–		–	–	–	–	
No	24 (7%)	23 (13%)	6 (19%)			32 (12%)	54 (17%)	7 (11%)			–	–	–	–		–	–	–	–	
Missing	*1*	*0*	*1*			*4*	*2*	*1*			–	–	–	–		–	–	–	–	
**pT-stage**				*0.007*	*0.142*				*.199*	*.284*				*0.161*	*0.399*				*<0.001*	*0.438*
pT0-pT2	145 (42%)	53 (31%)	6 (18%)			104 (43%)	69 (26%)	19 (33%)			305 (27%)	155 (21%)	68 (23%)			589 (43%)	291 (26%)	101 (29%)		
pT3-pT4	197 (58%)	119 (69%)	27 (82%)			140 (57%)	193 (74%)	38 (67%)			810 (73%))	587 (79%)	224 (77%)			792 (57%)	810 (74%)	253 (72%)		
Missing	*1*	*1*	*0*			*28*	*53*	*7*			*3115*	*1802*	*1002*			*30*	*12*	*11*		
**pN stage**				*0.01*	*0.268*				*.020*	*.333*				*<0.001*	*0.801*				*0.006*	*0.600*
pN0	162 (47%)	59 (34%)	8 (24%)			136 (56%)	120 (46%)	22 (39%)			1242 (36%)	570 (25%)	289 (26%)			693 (50%)	442 (40%)	151 (41%)		
pN+	180 (53%)	114 (66%)	25 (76%)			108 (44%)	143 (54%)	35 (61%)			2248 (64%)	1678 (75%)	833 (74%)			704 (50%)	665 (60%)	213 (59%)		
Missing	*1*	*0*	*0*			*28*	*52*	*7*			*734*	*296*	*172*			*14*	*6*	*1*		
**TRG**									*.066*	*<.001*						***(n = 1506)***	***(n = 1255)***	***(n = 411)***	*0.094*	*0.002*
TRG1-2	–	–	–			56 (26%)	24 (10%)	21 (38%)			–	–	–			190 (35%)	98 (28%)	47 (44%)		
TRG3-4	–	–	–			162 (74%)	209 (90%)	34 (62%)			–	–	–			347 (65%)	252 (72%)	60 (56%)		
Missing	–	–	–			*54*	*82*	*9*			–	–	–			*969*	*905*	*304*		

a Additional factors of which data is not shown: allocated treatment arm (n.s.). Dif-GC = diffuse type gastric cancer; GEJ = gastroesophageal junction; Int. GC = intestinal type gastric cancer; Muc. GC = mucinous type gastric cancer; IQR = interquartile range; *P* = *p* value; TRG = tumor regression grade.

**Figure 3.**
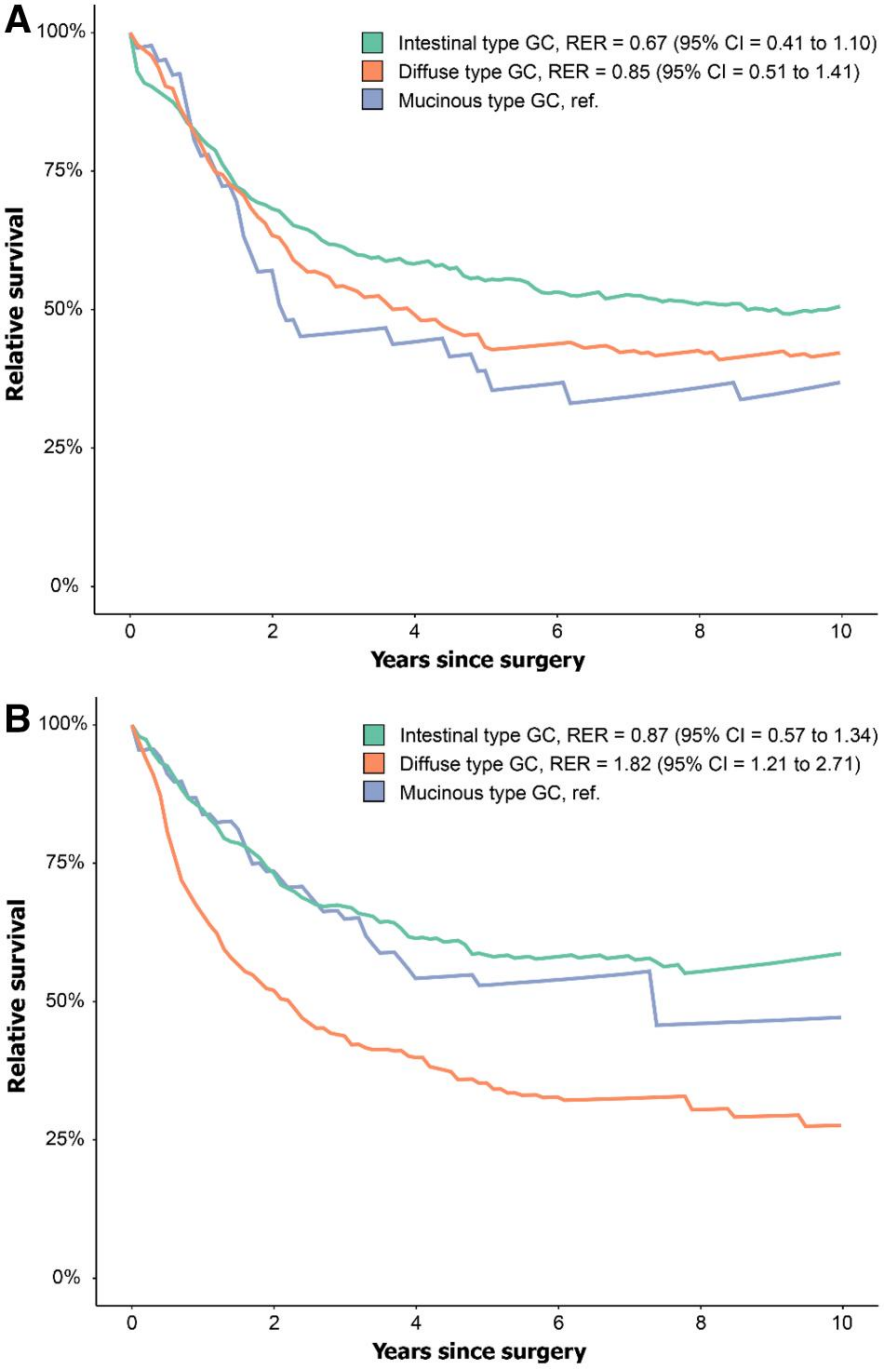
Relative survival in A) Surgery alone test cohort. B) Chemotherapy test cohort. GC = gastric cancer; RER = relative excess risks of death.

Patients with muc-GC showed a lower 5-year relative survival of 39% than 55% in patients with int-GC (RER 0.67, 95% CI = 0.41 to 1.10) and 43% in dif-GC (RER 0.85, 95% CI = 0.51 to 1.41); however, this is not statistically significant (Figure 3, A).

#### Chemotherapy test cohort

In the CT_test_ cohort, 64 of 651 evaluable GCs were muc-GC (10%), 272 int-GC (42%), and 315 dif-GC (48%) (Figure 2). The median follow-up was 3.5 years (IQR = 1.0-6.0). As shown in Table 1, median age at diagnosis and the distribution of sexes were similar to the SA_test_ cohort. Muc-GC was localized in the distal part of the stomach in 45% of patients, compared with 29% in int-GC (*P* = .099) and 36% in dif-GC (*P* < .001).

Gastrectomy with curative intent was performed in 89% of muc-GC, 88% of int-GC, and 83% of dif-GC patients. Positive lymph nodes (ypN+) were more frequently observed in patients with mucinous type and dif-GC than in int-GC patients (*P* = .020). TRG was available for 506 patients (78%). A (near-) complete response (TRG1-2) was significantly more frequent in 38% of muc-GC patients, compared with 26% of int-GC (*P* = .066) and 10% of dif-GC patients (*P* < .001).

As shown in Figure 3, B, patients with a muc-GC and int-GC had a similar 5-year RS of 53% and 58%, respectively (RER 0.87, 95% CI = 0.57 to 1.34), which was significantly higher compared with 35% in patients with dif-GC (RER 1.82, 95% CI = 1.21 to 2.71).

#### Surgery alone validation cohort

Of the 8468 patients diagnosed with GC before the year 2007, 8062 patients (95%) did not receive any pre- or postoperative chemo- or radiotherapy and could therefore be included in the SA_validation_ cohort. Among them were 1294 muc-GC (16%), 4224 (52%) int-GC, and 2544 (32%) dif-GC patients (Figure 2). The median follow-up was 1.9 years (IQR = 0.7-6.7). The distribution of age and sex was similar to the other cohorts (Table 1). Muc-GC was most often diagnosed in the distal part of the stomach (52%). Lymph node metastases (pN+) were more common in muc-GC compared with int-GC (*P* < .001), similar to dif-GC (*P* = .801).

**Figure 4.**
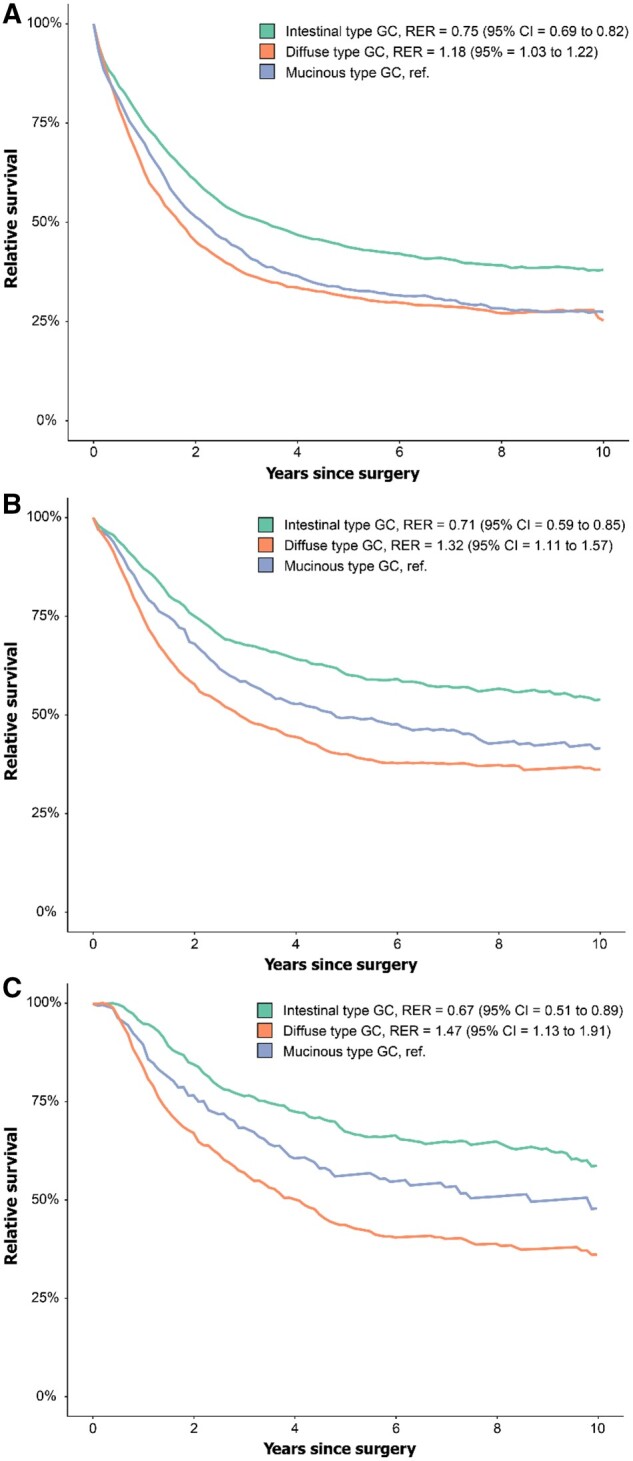
Relative survival in A) Surgery alone validation cohort. B) Chemotherapy validation cohort. C) Per-protocol perioperatively treated patients in the chemotherapy validation cohort. GC = gastric cancer; RER = relative excess risks of death.

When adjusted for sex and age differences in the general population, patients with muc-GC had a slightly higher 5-year relative survival of 31% than 29% in dif-GC (RER 1.18, 95% CI = 1.03 to 1.22), which was significantly lower compared with 42% in int-GC (RER 0.75, 95% CI = 0.69 to 0.82) (Figure 4, A).

#### Chemotherapy validation cohort

In total, 2889 (45%) of the 6419 patients diagnosed with GC between 2007 and 2019 underwent at least one cycle of preoperative chemotherapy and no radiation or targeted therapy. In this CT_validation_ cohort, 365 (13%) of the 2889 evaluable GCs were muc-GC, 1411 (49%) int-GC, and 1113 (38%) dif-GC, as shown in Figure 2. The median follow-up was 2.4 years (IQR = 1.1-5.6). Median age at diagnosis and sex followed a similar distribution as in the other cohorts (Table 1). Muc-GC was diagnosed in the gastroesophageal junction/cardia in 45% of patients.

**Figure 5.**
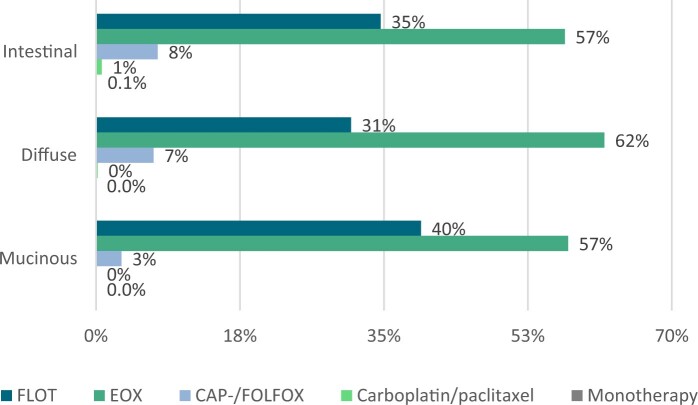
Chemotherapy regimen administered in each histological subtype in the chemotherapy validation cohort. FLOT = 5-fluorouracil or capecitabine, oxaliplatin, and docetaxel; EOX = epirubicin, oxaliplatin or cisplatin, and capecitabine or 5-fluorouracil; CAP-/FOLFOX = capecitabine or 5-fluorouracil and oxaliplatin; Monotherapy: capecitabine or 5-fluorouracil.

Details on the chemotherapy regimen were available for 1309 patients (45%). No significant differences in chemotherapy regimens were found between muc-GC, int-GC, and dif-GC patients (*P* = .273, Figure 5).

Advanced tumor stage (pT3-pT4) and lymph node metastases (pN+) were more frequently diagnosed in muc-GC compared with int-GC (*P* < .001 and *P* = .006, respectively), similar to dif-GC (*P* = .438 and *P* = .600, respectively). Data on TRG could be extracted from the pathology files of 994 of the 3127 patients (32%) who underwent preoperative chemotherapy because the majority of pathology reports did not specifically mention information on the TRG according to Mandard. In muc-GC, 44% of patients had (near-) complete response (TRG1-2) compared with 35% in int-GC (*P* = .094) and 28% in dif-GC (*P* = .002).

The 5-year relative survival of 48% in muc-GC patients was worse than 59% in int-GC patients (RER 0.71, 95% CI = 0.59 to 0.85), but significantly better than 38% in dif-GC patients (RER 1.32, 95% CI = 1.11 to 1.57) (Figure 4, B). This effect was even more pronounced in the subgroup of patients who underwent both preoperative and postoperative chemotherapy (RER 1.47, 95% CI = 1.13 to 1.91, Figure 4, C).

## Discussion

In this study, we presented the clinicopathological characteristics and survival of resectable muc-GC in 2 randomized phase III clinical trials. In one trial, patients were treated with surgery only, whereas in the other, patients underwent perioperative treatment. We thereafter validated these results using data of a large nationwide validation cohort. All combined, our findings indicate that patients with muc-GC tend to have a similar or only minimally better prognosis than those with dif-GC, which is worse than those with int-GC, when treated with surgery alone. After preoperative chemotherapy however, muc-GC showed a more favorable histopathological response and a significantly superior relative survival compared with dif-GC, and these findings were independent of age and sex. This positive effect of chemotherapy on survival in muc-GC was even more pronounced in patients receiving both preoperative and postoperative chemotherapy.

To our knowledge, this is the first study investigating the pathological response to preoperative chemotherapy among patients with muc-GC. In the surgery alone setting, our findings are in line with multiple studies that showed more advanced tumor stages (11, 13, 28) and worse survival (12-14, 28) of muc-GC compared with non-mucinous GC. Combining both int-GC and dif-GC as non-mucinous may, however, be misleading, as it is well established that these histopathological subtypes are distinct entities with differential clinical behavior and prognosis (2, 4, 6). Therefore, we divided non-mucinous GC into int-GC and dif-GC according to Lauren’s classification to provide clearer insight into the clinicopathological characteristics of patients with muc-GC. With this approach, we were able to show that the relative prognosis of muc-GC has improved compared with dif-GC since the introduction of perioperative chemotherapy.

In colorectal cancer, the mucinous phenotype has been associated with microsatellite instability (MSI-high) and a poor response to chemotherapy (19, 20, 30, 31). For gastric cancer, the evidence regarding MSI-high enrichment in the mucinous phenotype is conflicting (29, 32, 33). In a previous analysis of the D1/D2 and CRITICS trial, such an association was not found; only 4 out of 62 (6%) muc-GCs were MSI-high, similar to the entire included study population of 626 patients (5.5%) (34). Remarkably, a previous analysis of the MSI-high tumors in our CT_test_ cohort only showed histopathological response to chemotherapy in case of a mucinous differentiation (34). Our results demonstrate that this chemotherapy response can be observed not only in the specific subset of mucinous MSI-high cases but applies to all muc-GCs.

The standard treatment for resectable GC differs globally. In Europe and the United States, the standard treatment consists of D2 gastrectomy with perioperative fluorouracil plus leucovorin, oxaliplatin, and docetaxel (FLOT) (35, 36). In Asia, however, patients are treated mostly with postoperative chemotherapy after a D2 gastrectomy (37-39). Our results showed a significantly favorable histopathological response to chemotherapy in muc-GC compared with dif-GC in both the clinical trial and real-world setting, which was even more pronounced in the perioperative treated population.

Personalizing treatment for patients with GC based on their histological subtype may be challenging because this requires an accurate diagnosis on pretreatment biopsies. According to the WHO classification tumors must consist of more than 50% extracellular mucus to be classified as mucinous. Mucinous tumors often exhibit mucinous lakes primarily in the deeper layers of the gastric wall, potentially leading to lower mucus content (<50%) in biopsies. In our study, muc-GC determination therefore primarily relied on resection specimens, where the mucinous phenotype is more accessible. This could be limiting in clinical decision-making because a previous study showed that muc-GC as determined on the resection specimen was classified as such in only 30% to 50% of matched biopsies (40). However, extracellular mucus in pretreatment biopsies has been identified as an indicator for mucinous phenotype classification in the resection specimen in more than 70% of the mucinous tumors included in the study of Biesma et al. (34) Additionally, Cai et al. showed that the TNM tumor stages and prognosis of 514 GCs with a mucinous component comprising less than 50% were similar to those of 176 muc-GCs included in their analysis (11). The potential utility of extracellular mucus presence in pretreatment biopsies as an indicator for the mucinous phenotype in the resection specimen should be explored in future research.

It is important to acknowledge the limitations in our study design. First, our study approach did not enable a direct comparison between surgery alone and chemotherapy, because studies on this comparison are scarce and only include limited mucinous type tumors. However, using both clinical trial and nationwide data provided unique insight into how mucinous tumors have responded to the various standard treatments over time, compared with the intestinal and diffuse subtypes. Whether recent advancements in treatment, such as immunotherapy, will also hold promise for patients with muc-GC needs to be investigated in future studies. Another limitation may be that we were unable to exclude patients included in the CT_test_ cohort from the nationwide CT_validation_ cohort due to privacy regulations. However, while classification of both the histological subtype and TRG in the CT_test_ cohort were assessed at central pathology review, the CT_validation_ cohort relied solely on pathology reports. Data of the patients included in the test cohort could therefore still be classified as “unknown” or “missing data” in the validation cohort. Last, the histopathological subtype determination relied on syntax classification based on pathology reports in the nationwide validation cohorts. Although the histopathological subtype classification syntax achieved 96% accuracy, some intestinal and diffuse type tumors might have been classified as mucinous, potentially diluting the muc-GC subgroup. This could account for the slightly increased incidence of muc-GC in the validation cohorts compared with test cohorts and the more pronounced benefit of chemotherapy observed in mucinous gastric cancer within the clinical trial setting of the CT_test_ cohort.

Intestinal and diffuse type GC have long been established as important GC subtypes, differing with respect to stage of disease, metastatic patterns, response to chemotherapy, and survival (1-4). In the current study, 14% of all GCs were of the mucinous phenotype. Therefore, we propose an adjusted Lauren classification where mucinous type GC is recognized alongside intestinal and diffuse type GC to be used in both future clinical studies that stratify for histopathological subtype and in daily clinical practice. Acknowledgment of the mucinous subtype might help clinicians in personalized treatment planning because we showed the highest (near-) complete histopathological response rates in patients with mucinous type GC after preoperative chemotherapy, which was even more pronounced when patients were also treated with postoperative chemotherapy.

## Supplementary Material

djae227_Supplementary_Data

## Data Availability

The data that support the findings of this study are available on request from the corresponding author, the Netherlands Cancer Registry and Dutch Pathology Registry.
