# Supplementary material for: Histopathological response to chemotherapy and survival of mucinous type gastric cancer
Source: J Natl Cancer Inst. 2024 Sep 14;117(2):253–61. doi: 10.1093/jnci/djae227 (PMC11807439; doi:10.1093/jnci/djae227)
Supplement: djae227_Supplementary_Data [file djae227_supplementary_data.pdf]

Supplementary Material

Supplementary table 1. Terms used in histological subtype determination syntax for the validation cohorts. All negative and diminutive terms of the abovementioned, such as “no signet ring cells” or “little mucus secretion”, and terms with a distinct alternative meaning, such as “intestinal metaplasia”, were filtered by the syntax.

Intestinal	Diffuse	Mucinous
intestinal	diffuse	mucinous
glandular tubes	signet ring	mucin-forming
tubes	linitis	mucin formation
tubular	loose cells	colloid
tube-forming	scattered cells	mucus secretion
tube formation	scattered tumor cells	
tube structure	solitarily located	
tubulovillous	solitary tumor cells	
cribriform	solitary	
papillary	dissociative groups and solitary cells	
well-differentiated	loose atypical epithelial cells	
	disordered pattern	

Supplementary table 2. Terms used in syntax to determine tumor regression grade according to Mandard (TRG) for the validation cohorts.

TRG1	TRG2	TRG3	TRG4	TRG5
TRG 1	TRG 2	TRG 3	TRG 4	TRG 5
TRG I	TRG II	TRG III	TRG IV	TRG V
T0	Near complete response			No response
Complete response				

Supplementary table 3. Pathological AJCC/UICC TNM classifications of primary tumor (pT) since 1997.

Depth of invasion	TNM5 (1997)	TNM6 (2002)	TNM7 (2009)	TNM8 (2017)
**Mucosa**	pT1	pT1	pT1a	pT1a
**Submucosa**	pT1	pT1	pT1b	pT1b
**Muscularis propria**	pT2	pT2a	pT2	pT2
**Subserosa**	pT2	pT2b	pT3	pT3
**Serosa**	pT3	pT3	pT4a	pT4a
**Adjacent structures**	pT4	pT4	pT4b	pT4b
